# Prevalence and Types of Gross Lung Lesions in Cattle Slaughtered at the Gondar ELFORA Abattoir: A Cross‐Sectional Study

**DOI:** 10.1155/vmi/7539360

**Published:** 2026-08-02

**Authors:** Solomon Tsegaye, Birhan Getnet, Amare Bihon Asfaw

**Affiliations:** ^1^ Department of Veterinary Medicine, Woldia University, Weldiya, Ethiopia, wldu.edu.et

**Keywords:** abattoir, bovine, Gondar, gross examination, lung lesion

## Abstract

**Background and aim:**

The respiratory system, particularly the lungs, is highly vulnerable to various diseases due to the inhalation of contaminated air and the aspiration of drenches. This study aimed to determine the prevalence of gross lung abnormalities and their economic impact in cattle slaughtered at the Gondar ELFORA abattoir.

**Materials and methods:**

A cross‐sectional study was used in the study period between December 2023 and June 2024. Systematic random sampling was used to examine every fourth lung hung on the rail. A total of 382 lung samples were examined by visual inspection, palpation, and incision and analysed using descriptive statistical analysis, including calculation of prevalence and the χ^2^ test.

**Result:**

The study showed that 231 (60.45%) of the lungs examined had one or more lesions. The prevalence of specific lung conditions was as follows: emphysema (18.06%; *p* value < 0.001), hydatid cyst (13.87%; *p* value < 0.001), atelectasis (7.33%; *p* value < 0.001), pulmonary abscess (6.54%; *p* value < 0.001), haemorrhage (5.75%; *p* value < 0.001), fibrosis (3.14%; *p* value = 0.004), calcification (2.87%; *p* value = 0.036), oedema (2.6%) and lung worm infestation (0%). Hydatidosis and emphysema were significantly influenced both by body condition and age (*p* < 0.05). Cattle with thin body conditions showed significant associations with both atelectasis (*p* = 0.029) and abscess (*p* = 0.001). In contrast, there was no significant association between age and atelectasis (*X* ^2^ = 1.4595; *p* = 0.227) and abscess (*X* ^2^ = 2.6145; *p* = 0.106). Of the affected lungs, 176 were condemned due to aesthetic concerns and suspected zoonotic risks, representing a total rejection rate of 46%. The estimated annual economic loss due to lung rejection was approximately $11,836.95 (1,850,115.30 ETB) per year.

**Conclusion:**

During the study period, nearly half of the lungs of slaughtered cattle were rejected due to zoonotic concerns and aesthetic values. The substantial rate of pulmonary lesions with a predominant rate of hydatidosis and emphysema resulted in a considerable rejection rate, implicating a critical public health concern. The significant rate of economic loss from this abattoir with low slaughter volume could indicate huge economic loss at the country level and the urgency of improving herd health management.

## 1. Introduction

Gross pulmonary lesions are lung lesions and abnormalities that can be identified using naked eyes. The pathological abnormalities in the lungs, lung parenchyma, and associated lymph nodes commonly occurring as pneumonia, hydatid cyst, emphysema, atelectasis and others are detected during post mortem inspection [1].

The respiratory system is highly prone to infection due to its direct communication with the external environment. The anatomical and functional complexities of the respiratory system play a major role in determining how the respiratory tract reacts to damage and the patterns of diseases that follow. Because pulmonary lesions can interfere with pulmonary functions, particularly the oxygenation of blood and delivery of oxygen, farmers have reported decreased productivity and significant financial losses [2–4].

Many factors cause lung condemnation, including oedema, emphysema, pneumonia, fibrosis, calcification, atelectasis and hydatidosis [5]. Among the common pathologies, cystic hydatidosis, often known as hydatidosis, is one of the most widespread zoonoses globally, affecting both domestic animals and humans in different regions [6].

In damaged lungs, dystrophic calcification manifests as a localised, well‐organised accumulation of the calcium salt hydroxyapatite, typically following an inflammatory process. The metabolic lung illness known as metastatic pulmonary calcification features the accumulation of calcium in the pulmonary parenchyma. It most frequently happens in conjunction with illnesses that cause hypercalcemia, either directly or indirectly [7].

Pulmonary oedema is commonly observed in conditions affecting the systolic and diastolic function of the left ventricle, such as acute myocarditis and various nonischemic cardiomyopathic conditions, as well as acute myocardial infarction. One of the most frequent anomalies in chest imaging, atelectasis continues to present diagnostic challenges on a regular basis [8].

Many of the respiratory diseases in cattle have zoonotic and aesthetic implications that often result in the total rejection of the lungs or in partial trimming of the lungs, resulting in high economic loss in many of the abattoirs in Ethiopia [6]. From a One Health perspective, lung lesions not only resulted in direct economic burden but also increased the zoonotic risks, such as hydatidosis transmission to humans.

In Ethiopia, reports have indicated that hydatidosis is the primary cause of lung condemnation at abattoirs. Given its zoonotic importance, this public health concern requires urgent One Health strategies [6]. Most of the previous studies in Ethiopia have concentrated on the investigation of the prevalence and financial implications of gross pulmonary lesions, mostly in cattle [9]. Over the past five or more years, there have been no comprehensive studies on gross lung lesions in cattle slaughtered at Ethiopian abattoirs, despite the studies done on specific lesions like bovine lung hydatidosis [5, 10, 11] and calcification [12].

The Gondar ELFORA abattoir was selected as a surveillance site for pulmonary lesions in cattle due to several reasons, including cattle from different agroecological zones coming to this abattoir from‐high risk areas of hydatidosis and the slaughter of a mixed cattle population originating from faraway places. This mix‐up of different herds from different locations brought the advantage of monitoring the disease prevalence in the surrounding areas of one centre. Therefore, the objective of this study was to determine the prevalence and major types of gross lung lesions in cattle slaughtered at Gondar ELFORA abattoir and its economic impact.

## 2. Materials and Methods

### 2.1. Study Area

The study was carried out at the Gondar city’s Elfora slaughterhouse. It is located at latitude 12°36′ N and longitude 37°28′ *E*, and its elevation is 2133 m above sea level. According to the Central Statistical Agency of Ethiopia (CSA) [13], there are an estimated 3.2 million cattle, 1.3 million sheep, 1.8 goats, 33,000 horses, 13,000 mules and 400,000 donkeys living in Gondar town. There is a range of 12.5°C–32°C in temperature and 880–1772 mm of annual rainfall.

### 2.2. Study Design

A cross‐sectional study was conducted on cattle lungs at the Gondar Municipality abattoir to investigate the total prevalence and characteristics of gross pulmonary lesions in cattle. Data were collected between December 2023 and June 2024 through palpation and inspection, which were performed immediately after slaughter.

### 2.3. Study Population

Apparently healthy cattle slaughtered at the abattoir were the study population. The animals were male local cattle breeds sourced from Gondar city and immediate vicinities such as Demibia, Wegera, Aymba and Metema, where extensive production systems operate.

### 2.4. Sampling Methods

Systematic random sampling was used to examine every fourth lung hung on the rail. The every fourth lung sampling method was chosen due to the high number of cattle slaughtered during the holiday periods exceeding the capacity to examine every animal and also allowing representative coverage of the sample line. The Gondar ELFORA abattoir had a daily slaughter capacity of 20–30 cattle, but this number exceeded 40 cattle during holidays.

Lungs were excluded if an acute systemic illness was identified during antemortem inspection, as such cases indicate non‐representative end stage disease. Additionally, lungs that were partially or fully condemned due to injury, damage or incomplete evisceration were excluded from the sample.

### 2.5. Antemortem and Postmortem Procedures

The investigation followed standard protocols provided by the Ministry of Agriculture, Ethiopia [14], beginning with antemortem inspection within 24 h of abattoir stay. During antemortem, cattle were examined both at rest and in motion to identify any respiratory disease signs, such as breathing difficulties, nasal discharge and coughing. During the postmortem inspection, a detailed examination of the lungs was performed through visual inspection, palpation and incision.

### 2.6. Body Condition Scoring

Body condition scoring was performed and graded as thin, moderate or fat based on an evaluation of rib, pelvic and spine bone visibility in line with recommendations given by the Oklahoma Cooperative Extension Service [15]. If all three bones mentioned were visible, it was regarded as thin; if ribs were not prominent and the spine was smooth, it was regarded as moderate; and if all three bones were hidden and there was an obvious thick fat deposit, it was regarded as fat.

### 2.7. Age Determination

Dentition‐based age determination was made to categorise beef for slaughter as adult and young. Based on Japro’s explanation [16], age below 6 years and above 6 years were taken as young and adult, respectively.

### 2.8. Sample Size Determination

An overall prevalence of 53.6% for gross pulmonary lesion, as reported by Mekuriaw [17] at the Gondar Elfora abattoir, was used for sample size determination. This previous study was selected because it provides relevant baseline data from the same site. The sample size calculation assumed a 95% confidence level with a *Z* score of 1.96 and a 5% margin of error. Therefore, sample size was determined by substituting the expected prevalence of 53.6% and a 5% margin of error.

(1) N=1.962 Pexp 1−Pexpd2,

where *N* is the required sample size. *P*_exp_ is the expected prevalence, and *d* is required precision. By using the above formula, the required sample size was calculated to be the total sample, 382.

### 2.9. Sample Collection and Processing

Postmortem examination was performed immediately after slaughter using palpation, ocular inspection and incision to identify lung lesions. Upon incision, gross abnormalities including the lesions’ location, size and gross appearance were recorded. A total of 382 lung samples were examined for the presence of gross pulmonary lesions.

### 2.10. Lesion Identification Criteria

The identification of lesions was made based on the morphological criteria. Emphysema was characterised by inflated lung tissue with a pale appearance, whereas atelectasis was identified by a depressed, dark red area and firmness upon palpation. Hydatid cyst was identified as fluid‐filled tissue, while a pulmonary abscess was identified as a small, focal and encapsulated lesion containing pus. In cases of haemorrhage, the tissue was found as bright or dark red coloured. Calcification was identified by small, hard and whitish or yellowish granule presence during incision [18].

### 2.11. Data Collection and Analysis

Animals originating from different areas were inspected, and all abnormalities observed during antemortem inspection or on the lung post slaughter were recorded in a note book. All collected data were organised and entered in to MS Excel, then exported to Stata version 17. Descriptive statistics and χ^2^ test were used to analyse the data.

## 3. Results

### 3.1. Pulmonary Lesions

Out of the 382 slaughtered cattle examined, 220 (57.8%, 95% CI: 52.6–62.5) presented with gross pulmonary lesions. The most common conditions identified were pulmonary emphysema, 69 (18.06%, 95% CI: 14.5–22.3); hydatidosis, 53 (13.87%, 95% CI: 10.7–17.7); atelectasis, 28 (7.33%, 95% CI: 5.1–10.4); pulmonary abscesses, 25 (6.54%, 95% CI: 4.4–9.5); pulmonary haemorrhage, 22 (5.75%, 95% CI: 3.8–8.6); fibrosis (3.14%, 95% CI: 1.8–5.5) and calcification (2.87%, 95% CI: 0.04–1.8). Pulmonary emphysema was the most prevalent lung lesion, with hydatidosis being the second most frequent finding (Table 1).

**TABLE 1:** Prevalence of cattle lungs lesions.

Type of lesion	Total examined	Lesion positive	Prevalence (%)	*X* ^2^ (*p* value)	95% CI
Pulmonary emphysema	382	69	18.06	55.04 (0.000)	14.5–22.3
Pulmonary hydatidosis	382	53	13.87	40.22 (0.000)	10.7–17.7
Pulmonary atelectasis	382	28	7.33	19.75 (0.000)	5.1–10.4
Pulmonary abscesses	382	25	6.54	17.48 (0.000)	4.4–9.5
Pulmonary haemorrhage	382	22	5.75	15.25 (0.000)	3.8–8.6
Pulmonary fibrosis	382	12	3.14	8.1 (0.004)	1.8–5.5
Pulmonary calcification	382	11	2.87	4.38 (0.036)	0.04–1.8
Total		220	57.8%		52.6–62.5

The analysis in Table 2 revealed hydatidosis and emphysema were significantly influenced by both body condition and age (*p* < 0.05). Older cattle and thin cattle were significantly affected by hydatidosis (prevalence = 18.53% and 26.6%) and emphysema (prevalence = 26.72% and 37.6%). Cattle with thin body condition showed significant associations with both atelectasis (*p* = 0.029) and abscess (*p* = 0.001). In contrast to abscess, there was no significant association between age and atelectasis (*X* ^2^ = 1.4595; *p* = 0.227) and abscess (*X* ^2^ = 2.6145; *p* = 0.106).

**TABLE 2:** The association of lung lesions with body conditions and age group.

Lesion/abnormality	Factor	Categories	No. of cattle examined	Positive cases (%)	*X* ^2^‐value	*p* value
Hydatidosis	Age	< 6 years	150	10 (6.7%)	10.737	0.001
		> 6 years	232	43 (18.53)		
	Body condition	Thin	109	29 (26.6)	26.982	0.000
		Moderate	230	15 (6.52)		
		Fat	43	9 (20.93)		

Emphysema	Age	< 6 years	150	7 (4.67)	29.95	0.000
		> 6 years	232	62 (26.72)		
	Body condition	Thin	109	41 (37.6)	39.86	0.000
		Moderate	230	22 (9.56)		
		Fat	43	6 (13.95)		

Atelectasis	Age	< 6 years	150	14 (9.33)	1.4595	0.227
		> 6 years	232	14 (6.03)		
	Body condition	Thin	109	14 (12.84)	7.084	0.029
		Moderate	230	11 (4.78)		
		Fat	43	3 (6.98)		

Abscess	Age	< 6 years	150	6	2.6145	0.106
		> 6 years	232	19		
	Body condition	Thin	109	15 (4.59)	13.1376	0.001
		Moderate	230	9 (3.91)		
		Fat	43	1 (2.33)		

#### 3.1.1. Pulmonary Emphysema

Among the gross pulmonary lesions observed, emphysema occurred with the highest frequency (18.06%) (Table 3).

**TABLE 3:** Prevalence of pulmonary emphysema.

Types of lobes	Lesion positive	Prevalence (%)
Right cranial	4	1.04
Right medial	54	14.14
Right caudal	2	0.52
Right accessory	7	1.83
Left caudal	2	0.52
Total	69	18.06

#### 3.1.2. Pulmonary Haemorrhage

Among gross pulmonary lesions observed, the prevalence of pulmonary haemorrhage was 5.75%; the left cranial lobe was the site most frequently affected by pulmonary haemorrhage (Table 4).

**TABLE 4:** The prevalence of pulmonary haemorrhage.

Types of lobes	Lesion positive	Prevalence (%)
Right cranial	4	1.05
Right medial	2	0.52
Right caudal	3	0.79
Left cranial	9	2.36
Left caudal	4	1.04
Total	22	5.75

#### 3.1.3. Pulmonary Hydatidosis

The prevalence of pulmonary hydatidosis was 13.87%. The right caudal lung lobe was affected most frequently by this lesion (Table 5).

**TABLE 5:** The prevalence of hydatidosi**s**.

Types of lobes	Frequency	Prevalence%
Right medial	16	4.19
Right caudal	36	9.42
Left caudal	1	0.26
Total	53	13.87

#### 3.1.4. Pulmonary Atelectasis

The prevalence of pulmonary atelectasis was 7.33%; the right medial lung lobe was the most affected site by this condition (Table 6).

**TABLE 6:** The prevalence of pulmonary atelectasi**s**.

Types of lobes	Frequency	Prevalence%
Right cranial	1	0.26
Right medial	20	5.24
Right caudal	1	0.26
Left caudal	6	1.57
Total	28	7.33

#### 3.1.5. Pulmonary Fibrosis

The prevalence of pulmonary fibrosis was found to be 3.14%, mostly frequently affecting the right lung lobe (Table 7).

**TABLE 7:** The prevalence pulmonary fibrosi**s**.

Types of lobes	Lesion positive	Prevalence%
Right medial	1	0.26
Right cranial	1	0.26
Right caudal	6	1.57
Left cranial	1	0.26
Left caudal	3	0.79
Total	12	3.14

#### 3.1.6. Pulmonary Calcification

The prevalence of pulmonary calcification was 2.87%, with the right caudal lung lobe mostly affected by this condition (Table 8).

**TABLE 8:** The prevalence pulmonary calcification.

Types of lobes	Lesion positive	Prevalence%
Right cranial	1	0.26
Right medial	2	0.52
Right caudal	8	2.09
Total	11	2.87

#### 3.1.7. Pulmonary Abscess

The prevalence of pulmonary abscesses was 6.54%, with the right cranial lung lobe being the site most frequently affected by this condition (Table 9).

**Table 9:** The prevalence of pulmonary abscess.

Types of lobes	Lesion positive	Prevalence (%)
Right cranial	20	5.24
Right caudal	2	0.52
Right accessory	3	0.79
Total	25	6.54

### 3.2. Estimation of Economic Loss

The economic impact was estimated based on an expected rejection rate (46%), the market value of the lung ($4.7) and the minimum annual slaughter volume (15 animals per day; 5475 per year). As indicated in Table 10, the total annual financial loss was calculated to be $11,836.95 (1,850,115.30 ETB). Lungs were rejected based on the presence of extensive lesions, including abscesses of size > 1 cm, atelectasis with significant consolidation and extensive emphysema; additionally, all those positive for hydatid cysts were condemned. The financial loss due to lung condemnation was calculated based on the formula established by Ogunrinade [19].

(2) EL=∑srx∗Coy∗Roz,

where EL = annual economic loss due to lung condemnation;∑srx = annual cattle slaughter rate of the abattoir;  Coy = average cost of each lung;  Roz = condemnation rate of cattle lung.

(3) EL=∑srx∗Coy∗Roz,

EL = 5475 × 4.7 ∗ 0.46 = $11,836.95 (1,850,115.30 ETB).

**TABLE 10:** Economic loss associated with lung lesions.

Organ	Rejection rate	Rejected lung per year	Selling cost per lung	Total
Lung	46%	5475	$4.7	$11,836.95
			Yearly loss in Ethiopian Birr	1,850,115.30 ETB^∗^

^∗^ETB (Ethiopian Birr) with the current exchange rate of 1 $ = 156.30 ETB; the conversion rate was of May 18, 2026, published by the Commercial Bank of Ethiopia.

## 4. Discussion

The total prevalence of pulmonary lesions in the current study was significantly lower than the 96.6% prevalence at Hawassa [9], 86.6% at the Abergelle abattoir in Mekelle [20] and 89% at US feedyards [1], but it is higher than 58.3% at the Bishoftu ELFORA abattoir [21], 17.9% at Leon, Spain [22], and 11.26% at the Abu Simbel abattoir, Egypt [23].

The higher prevalence was attributed to the long‐distance transport from high‐risk highland sources, which suggests that proper control measures were not implemented in the research area. Furthermore, the variance shown could be attributed to the local agroecology, the region’s severe climate change or other management strategies. Certain illnesses are exclusive to a particular agroecological niche, where the pathogen or its intermediate host may find advantageous circumstances.

The prevalence of pulmonary emphysema observed in this study was lower than 31.25% at the Abergelle abattoir, Mekelle [20], and 20.4% at the Hawassa Municipal abattoir, Ethiopia [9]. On the other hand, emphysema prevalence was higher than the 5.7% rate at the Bishoftu ELFORA abattoir [21] and the 2.86% rate at the Butembo public slaughterhouse, Democratic Republic of the Congo [8].

This intermediate rate of pulmonary emphysema might have arisen from environmental factors and different management practices which caused variation in prevalence. A physical examination revealed that emphysema was more common than the other nine abnormalities, with haemorrhage, oedema, calcification, hydatidosis, abscess, fibrosis and lung worms representing the next most frequent findings. In addition to being associated with illnesses, pulmonary emphysema could also be caused by airflow obstruction or prolonged gasping during the dying process [10]. Furthermore, incorrect stunning, delayed slaughter after stunning and delayed hoisting after slaughter may also have contributed to the high percentage of lungs with emphysema.

Hydatidosis was the second most common gross lung lesion found at the Gondar ELFORA abattoir, which indicated a serious zoonotic risk that demanded a stronger One Health strategy and approach to control the disease.

The prevalence rate of hydatidosis was higher than 6.67% at the Abergelle international export abattoir [20] and 10.4% at the Bishoftu municipal abattoir [24], whereas it was lower than the prevalence of 15.63% at the Adama municipal abattoir [12], 17.9% at the Wolaita Sodo municipal abattoir [10], 20.75% at the Gimbichu municipal abattoir, Hadya [25], 22.5% at the Burayu municipal abattoir [26] and 16.54% at the Abergelle international export abattoir [27].

The peri‐urban setup of Gondar, with a high population of dogs and wild carnivores, could be the reason facilitating the transmission of the disease through offal access. Hydatid cysts were most commonly found on the right caudal lobes of the lungs. This might have been because the caudal lobes receive a greater volume of blood supply because of their larger size [28].

From a One Health perspective, such a high transmission and prevalence rate pose a higher occupational hazard to abattoir workers, as they might contract the disease through direct inhalation or skin contact with a ruptured cyst during evisceration.

The prevalence rate of pulmonary abscesses was higher than rates of 2.08% at the Abergelle international export abattoir [20], 2.86% at the Bishoftu export abattoir [21] and 0.52% at the Butembo public slaughterhouse, Democratic Republic of the Congo [8].

Aspiration of foreign objects, septic emboli (such as a ruptured hepatic abscess in cattle) and direct traumatic lung penetration are the primary causes of pulmonary abscesses. In this study, the higher rate of abscesses compared to those in other studies was an indicator of stress during transportation associated with long‐distance trekking of cattle from highland sources, which might promote aspiration pneumonia and septic emboli. From a One Health perspective, a high abscess rate signals a potential zoonotic risk for abattoir workers through inhalation and cutaneous contamination by pathogens that may lead to a pyogenic lung infection [29].

Atelectasis, which is the collapse of the lungs, in this research was also higher than many other studies in Ethiopia, such as the 5% prevalence at the Hawassa municipal abattoir [2], 1.25% at the Abergelle international export abattoir [20] and 2.6% at the Bishoftu export abattoir [21], whereas it was lower than the 15.56% prevalence in Assosa, Bale Robe and Mekelle [30]. The high altitude of Gondar could be the reason for the higher prevalence of atelectasis than in Hawassa, Abergelle and Bishoftu [31].

The prevalence of pulmonary haemorrhage was lower than the 6.7% prevalence found at the Babylon Abattoir [32], the 6.8% prevalence at the Bishoftu ELFORA export abattoir [21], and the 12.08% prevalence at the Abergelle international export abattoir [20]. On the other hand, the prevalence was higher than the 0.4% rate found at the Hawassa Municipal abattoir [9] and the 0.39% prevalence at the Butembo public slaughterhouse, Democratic Republic of the Congo [8].

Since this study lacked a histological examination, it might have masked chronic contributors like verminosis, which was a study limitation. In addition, inspection of the lungs using only naked eyes might have underreported subclinical or early‐stage infections, leading to underestimating the prevalence rate. This lung lesion could have been due to crowding and exhaustion from long treks [21]. The highland nature of the Gondar area might have provided a suitable environment for respiratory pathogens, which might have aligned with seasonal stressful conditions for animals.

This study found no lung worms. The massive deworming programmes undertaken in the region and the season during the study period could have caused zero prevalence of lung worms [33].

This study explored the frequency of lung calcification, which was 2.87%, while the prevalence of pulmonary fibrosis was 3.14%; both were higher than the findings of 2.3% for calcification and 1.4% for fibrosis prevalence rate at the Hawassa Municipal abattoir [9] but lower than the 6.25% prevalence of calcification at the Bishoftu ELFORA export abattoir [21].

The findings of calcification and fibrosis were modest but still prevalent in the study area and were mainly associated with Gondar highland dynamics, where subclinical trekking induced stress and atelectasis could have resulted in dystrophic calcification [3].

The study demonstrated that both thin body condition and older age significantly increased the prevalence of emphysema and hydatidosis. This trend aligned with other studies, like the research in Batna, Algeria, where older animals had a significantly higher prevalence (18.53%) compared to young animals (6.7%) [34]. In another similar study, at the Nekemte municipality abattoir, both age and body condition had a statistically significant association with the prevalence of hydatidosis [6]. The reason for the older cattle having been highly affected by hydatidosis might be due to the longer time exposure of older cattle to parasitic eggs from contaminated pastures. On the other hand, the significant association between poor body condition and hydatidosis could be attributed to the parasitic burden, which might have led to weight loss.

Chronic stress on older cattle emanating from environmental cyclic stress, such as seasonal dust or smoke from traditional cattle houses, might have been the reason why emphysema prevalence was high in older cattle [20].

The prevalence of atelectasis and abscess showed no significant statistical association with age. This finding was consistent with the study conducted at the Hawassa municipal abattoir where age has no significant association with the prevalence of atelectasis (*p* = 0.129) [9].

A pulmonary abscess showed a highly significant correlation with poor body condition (*p* = 0.001). Abscesses were mostly associated with chronic diseases where appetite might have been reduced for a longer time, which consequently resulted in poor body condition [35].

The study revealed valuable information about the pulmonary lesions at the Gondar ELFORA abattoir, despite certain limitations. These limitations, such as selecting exclusively male cattle for the sample, contributed to sex‐related bias, as males have higher endurance for trekking stress, which might increase the prevalence of atelectasis or emphysema. Data collection only during the dry season was also a limitation of this study, which might have influenced the worm burden and haemorrhage.

## 5. Conclusion

In conclusion, this study from the Gondar ELFORA abattoir revealed an overall prevalence of 57.8%, which was substantial and led to a huge economic loss due to lung rejection. It had an intermediate prevalence compared to the Hawassa abattoir and the Bishoftu export abattoir, which highlighted how long‐distance highland trekking, agroecological factors, and insufficient control measures could increase the rate of pathological conditions in cattle. Major lesions detected were emphysema, atelectasis, hydatidosis, abscess and pulmonary haemorrhage. Body condition score was strongly associated with the highly prevalent lesions. The pronounced prevalence of hydatidosis, which has zoonotic importance, signalled a threat to public health; therefore, advocating for One Health integration and intervention is crucial. Therefore, strengthening disease surveillance and regular deworming and vaccination programmes to reduce the prevalence of respiratory infections in cattle are recommended to be made mandatory. Educating farmers, abattoir workers and veterinarians on best practices for disease prevention, as well as a One Health intervention by launching community education on the safe disposal of offal, protecting abattoir workers from ruptured cysts and minimising dog access to infected offal. Improved transportation systems and parasite control should be taken as the primary roles of the One Health prevention approach.

## Author Contributions

Solomon Tsegaye: conceived and proposed the study, collected and analysed the data, and edited, proof‐read and finalised the manuscript.

Birhan Getnet: collected data, and wrote and reviewed the article.

Amare Bihon Asfaw: wrote and reviewed the article.

## Funding

This study did not receive any funds but is a part of thematic research at Woldia University, Ethiopia.

## Ethics Statement

The Ethical Review Board of Woldia University [reference number WDU/IRB/001/2024, approval date July 12/2024 G C.] evaluated and approved the study. As this research did not involve live animal handling or euthanasia, the ethical review committee confirmed that there was no animal welfare issue which needed to be addressed under the National Research Ethics Review Guidelines established by the Ministry of Science and Technology of the Federal Democratic Republic of Ethiopia in 2014. Additionally, we secured verbal consent from the abattoir to access for investigation.

## Conflicts of Interest

The authors declare no conflicts of interest.

## Data Availability

The datasets generated during and/or analysed during the current study are not publicly available due to ethical restrictions of our university but are available from the corresponding author on reasonable request.
